# Inspection plan for COVID-19 patients for Weibull distribution using repetitive sampling under indeterminacy

**DOI:** 10.1186/s12874-021-01387-7

**Published:** 2021-10-25

**Authors:** G. Srinivasa Rao, Muhammad Aslam

**Affiliations:** 1 Department of Statistics, University of Dodoma, PO. Box: 259, Dodoma, Tanzania; 2 Department of Statistics, Faculty of Science, King Abdulaziz University, Jeddah, 21551 Saudi Arabia

**Keywords:** Repetitive sampling plan, Traditional statistics, Indeterminacy, COVID-19, Average sample number

## Abstract

**Background:**

This research work is elaborated investigation of COVID-19 data for Weibull distribution under indeterminacy using time truncated repetitive sampling plan. The proposed design parameters like sample size, acceptance sample number and rejection sample number are obtained for known indeterminacy parameter.

**Methods:**

The plan parameters and corresponding tables are developed for specified indeterminacy parametric values. The conclusion from the outcome of the proposed design is that when indeterminacy values increase the average sample number (ASN) reduces.

**Results:**

The proposed repetitive sampling plan methodology application is given using COVID-19 data belong to Italy. The efficiency of the proposed sampling plan is compared with the existing sampling plans.

**Conclusions:**

Using the tables and COVID-19 data illustration, it is concluded that the proposed plan required a smaller sample size as examined with the available sampling plans in the literature.

## Background

It is broadly established that a huge number of COVID-19 cases are unnoticed worldwide. A rudimentary measure of population occurrence is the small part of positive cases for a given date in any country. On the other hand, this is subject to largely found that bias since tests are normally only ordered from suggestive cases, whereas a large proportion of infected people might show little symptoms or sometimes no symptoms for more details see [1]. Most governments are applying the mechanism of test randomly selected individuals to estimate the true disease occurrence in inhabitants in a particular locality. Nevertheless, when the disease occurrence is low and difficult to acquire from each patient/person by tests, under such situations we may use an acceptance sampling plan under indeterminacy. The health practitioners are paying attention to estimate the average number of deaths or ratio of deaths to the total number of COVID-19 death cases on daily basis, for the coming days, next week or month, etc. Reference [2]. In such a case, the health practitioners are paying attention to test the null hypothesis that the average number of deaths or ratio of deaths to the total number of COVID-19 death cases on daily basis is equal to the specified average number of deaths due to COVID-19 against the alternative hypothesis that the average number of deaths due to COVID-19 varies significantly. In this situation for testing of the hypothesis, practically it is difficult to record the average number of death for the whole year, whereas it is easy to record the daily basis and the average number of deaths can be obtained from the randomly selected days. The null hypothesis may be rejected if the daily average number of deaths due to COVID-19, state acceptance number of days, is more than or equal to the specified average number of deaths due to COVID-19 throughout the given number of days.

Many researchers have done studies on the time truncated life test for various distributions. Some of them are [3] developed the acceptance sampling plan for life tests: log-logistic models. Reference [4] derived acceptance sampling based on truncated life tests for generalized Rayleigh distribution. Reference [5] developed the acceptance sampling plans based on the generalized Birnbaum-Saunders distribution. Reference [6, 7] constructed the acceptance sampling plans for Birnbaum-Saunders and Burr XII distributions. References [8, 9] constructed acceptance sampling plans for extended exponential and generalized inverted exponential distributions. The details about the acceptance sampling plans can be seen in [10, 11]. The generalization of a single acceptance sampling plan namely repetitive sampling plan, [12] derived the decision rule of the repetitive acceptance sampling plan. The method of repetitive group acceptance sampling plan (RGASP) was first proposed by [13] for an attribute. Reference [14, 15] constructed the RASP for inverse Gaussian distribution and Burr type XII. Reference [16] developed generalized inverted exponential distributions. References [17–19] studied the repetitive sampling plan under different situations.

More details about the neutrosophic logic, their measure of determinacy, and indeterminacy are given by [20]. Numerous authors studied the neutrosophic logic for various real problems and showed its efficiency over fuzzy logic, for more details refer [21–26]. The idea of neutrosophic statistics was given using the idea of neutrosophic logic, [27–29]. The neutrosophic statistics give information about the measure of determinacy and measure of indeterminacy, see [30]. The neutrosophic statistics reduce to classical statistics if no information is recorded about the measure of indeterminacy. References [31–33] proposed the acceptance sampling plans using neutrosophic statistics [34]. proposed the time-truncated group plans for the Weibull distribution. Reference [35] worked on neutrosophic Weibull and neutrosophic family of Weibull distribution.

The existing sampling plans based on classical statistics and fuzzy philosophies do not give information about the measure of indeterminacy. Reference [36] worked on the single sampling plan using a fuzzy approach. Reference [37] discussed the effect of sampling error on inspection using a fuzzy approach. Reference [38] proposed a single plan using fuzzy logic. Reference [39] proposed the improved sampling plan using fuzzy logic. For details, the reader may refer to [40, 41]. To the best of our knowledge, there is no work on a time-truncated sampling plan for Weibull distribution under indeterminacy. In this paper, a repetitive acceptance sampling plan for Weibull distribution under indeterminacy is developed to testing the daily average deaths. We are anticipated the proposed sampling plan shows a fewer sample size as compared with the existing sampling plans for testing the daily average deaths.

In Section 2, we present an introduction of a repetitive acceptance sampling plan for Weibull distribution under indeterminacy. In Section 3, the proposed repetitive acceptance sampling plan under indeterminacy is compared with the single sampling plan proposed by [42]. The proposed sampling plan is illustrated using COVID-19 data belong to Italy, which was recorded from 1 April to 20 July 2020 in Section 4. Finally, the conclusions and future research works are established in Section 5.

## Methods

The repetitive acceptance sampling plan depends upon the truncated life test procedure is developed by [43–45]. The operational steps of this test are given as follows:

- Step 1: Draw a sample of size *n* from the lot. These samples can be put on a life test for a fixed time t_0_. Specify the average *μ*_0_ and indeterminacy parameter *I*_*N*_*ϵ*[*I*_*L*_, *I*_*U*_].
- Step 2: Accept *H*_0_ : *μ*_*N*_ = *μ*_0*N*_ if the daily average deaths in *c*_*1*_ days are more than or equal to *μ*_0_ (i.e., *μ*_0_ ≤ *c*_1_). If daily average deaths in *c*_*2*_ days are less than to *μ*_0_ (i.e., *μ*_0_ *>c*_*2*_) then we reject *H*_0_ : *μ*_*N*_ = *μ*_0*N*_ and terminate the test, where *c*_*1≤*_ *c*_*2*_.
- Step 3: If *c*_*1*_*< μ*_0_ *≤c*_*2*_ then go to step 1 and repeat the above experiment.

The above procedure of repetitive acceptance sampling plan (RASP) mainly depends on four characteristics those are *n, c*_*1*_, *c*_*2*_ and *I*_*N*_, where *I*_*N*_*ϵ*[*I*_*L*_, *I*_*U*_] is considered as the specified parameter and set according to the uncertainty level. RASP is nothing but the generalization of an ordinary single sampling plan under uncertainty. If *c*_*1*_ *= c*_*2*_ in RASP, it ultimately reduces to a single sampling plan under uncertainty. Suppose that *t*_0_ = *aμ*_0_ be the time in days, where *a* is the termination ratio. The lot acceptance probability is to be determined with the help of operating characteristic (OC) function for details see [13] and it is given by

1 $$L(p)=\frac{P_a(p)}{P_a(p)+{P}_r(p)};0<p<1$$

Here *P*_*a*_(*p*) is the probability of accepting *H*_0_ : *μ*_*N*_ = *μ*_0*N*_ and *P*_*r*_(*p*) is the probability of rejecting *H*_0_ : *μ*_*N*_ = *μ*_0*N*_, which are given by

2 $${P}_a(p)=\sum \limits_{i=0}^{c_1}\left(\begin{array}{*{20}l} n\\ {}i\end{array}\right){p}^i{\left(1-p\right)}^{n-i}$$

and

3 $${P}_r(p)=1-\sum \limits_{i=0}^{c_2}\left(\begin{array}{*{20}l} n\\ {}i\end{array}\right){p}^i{\left(1-p\right)}^{n-i}$$

where p is the probability of unreliability.

Therefore eq. (1) becomes

4 $$L(p)=\frac{\sum \limits_{i=0}^{c_1}\left(\begin{array}{*{20}l} n\\ {}i\end{array}\right){p}^i{\left(1-p\right)}^{n-i}}{\sum \limits_{i=0}^{c_1}\left(\begin{array}{*{20}l} n\\ {}i\end{array}\right){p}^i{\left(1-p\right)}^{n-i}+1-\sum \limits_{i=0}^{c_2}\left(\begin{array}{*{20}l} n\\ {}i\end{array}\right){p}^i{\left(1-p\right)}^{n-i}};0<p<1$$

The Weibull distribution under neutrosophic statistics is developed by [42] for the design of the sampling scheme plan for testing the average wind speed under an indeterminate environment.

Suppose that *f*(*x*_*N*_) = *f*(*x*_*L*_) + *f*(*x*_*U*_)*I*_*N*_; *I*_*N*_*ϵ*[*I*_*L*_, *I*_*U*_] be a neutrosophic probability density function (npdf) having determinate part *f*(*x*_*L*_), indeterminate part *f*(*x*_*U*_)*I*_*N*_ and indeterminacy interval *I*_*N*_*ϵ*[*I*_*L*_, *I*_*U*_]. Note that *x*_*N*_*ϵ*[*x*_*L*_, *x*_*U*_] be a neutrosophic random variable follows the npdf. The npdf is the generalization of pdf under classical statistics. The proposed neutrosophic form of *f*(*x*_*N*_)*ϵ*[*f*(*x*_*L*_), *f*(*x*_*U*_)] reduces to pdf under classical statistics when *I*_*L*_ =0. Based on this information, the npdf of the Weibull distribution is defined as follows.

5 $$f\left({x}_N\right)=\left\{\left(\frac{\beta }{\alpha}\right){\left(\frac{x_N}{\alpha}\right)}^{\beta -1}{e}^{-{\left(\frac{x_N}{\alpha}\right)}^{\beta }}\right\}+\left\{\left(\frac{\beta }{\alpha}\right){\left(\frac{x_N}{\alpha}\right)}^{\beta -1}{e}^{-{\left(\frac{x_N}{\alpha}\right)}^{\beta }}\right\}{I}_N;{I}_N\epsilon \left[{I}_L,{I}_U\right]$$

where *α* and *β* are scale and shape parameters, respectively. Note here that the proposed npdf of the Weibull distribution is the generalization of pdf of the Weibull distribution under classical statistics. The neutrosophic form of the npdf of the Weibull distribution reduces to the Weibull distribution when *I*_*L*_ =0. The neutrosophic cumulative distribution function (ncdf) of the Weibull distribution is given by

6 $$F\left({x}_N\right)=1-\left\{{e}^{-{\left(\frac{x_N}{\alpha}\right)}^{\beta }}\left(1+{I}_N\right)\right\}+{I}_N;{I}_N\epsilon \left[{I}_L,{I}_U\right]$$

The neutrosophic mean of the Weibull distribution is given by.

7 $${\mu}_N=\alpha \Gamma \left(1+1/\beta \right)\left(1+{I}_N\right);{I}_N\epsilon \left[{I}_L,{I}_U\right]$$

The null and alternative hypotheses for the daily average deaths are stated as follows:

$${H}_0:{\mu}_N={\mu}_{0N}\kern0.5em \mathrm{Vs}.{H}_1:{\mu}_N\ne {\mu}_{0N}.$$

Where *μ*_*N*_ is a true daily average death and *μ*_0*N*_ is the specified daily average deaths. Suppose that *t*_0*N*_ = *aμ*_0*N*_ be the time in days, where *a* is the termination ratio. The probability of the item will fail before it reaches the experiment time *t*_0*N*_ is defined as follows:

8 $${\displaystyle \begin{array}{*{20}c}{p}_N=1-\left\{{e}^{-{\left(\frac{t_{0N}}{\alpha}\right)}^{\beta }}\left(1+{I}_N\right)\right\}+{I}_N;{I}_N\epsilon \left[{I}_L,{I}_U\right]\\ {}=1-\left\{\exp \left(-{a}^{\beta }{\left({\mu}_N/{\mu}_{0N}\right)}^{-\beta }{\left(\Gamma \left(1/\beta \right)/\beta \right)}^{\beta }{\left(1+{I}_N\right)}^{\beta}\right)\left(1+{I}_N\right)\right\}+{I}_N\end{array}}$$

where *μ*_*N*_/*μ*_0*N*_ is the ratio of true average daily wind speed to specified average daily wind speed. Suppose that $\tilde{\alpha }$ and $\tilde{\beta }$ be type-I and type-II errors. The medical practitioners are interested to apply the proposed plan for testing *H*_0_ : *μ*_*N*_ = *μ*_0*N*_ such that the probability of accepting *H*_0_ : *μ*_*N*_ = *μ*_0*N*_ when it is true should be larger than $1-\tilde{\alpha }$ at *μ*_*N*_/*μ*_0*N*_ and the probability of accepting *H*_0_ : *μ*_*N*_ = *μ*_0*N*_ when it is wrong should be smaller than $\tilde{\beta }$ at *μ*_*N*_/*μ*_0*N*_ = 1. In order to find the design parameters *n, c*_*1*_, *c*_*2*_ and *I*_*N*_ for the proposed RASP, we consider two points on the OC function. In our approach, the quality level mainly depends on the ratio *μ*_*N*_/*μ*_0*N*_. This ratio is helpful for the producer to improve the lot quality. From in producer point of view, the probability of acceptance should be at least $1-\tilde{\alpha }$ at acceptable quality level (AQL), *p*_1*N*_. So, the producer demands the lot should be accepted at various levels of *μ*_*N*_/*μ*_0*N*_. Similarly, from in consumer point of view the lot rejection probability should not be exceeded $\tilde{\beta }$ at limiting quality level (LQL), *p*_2*N*_. The design parameters are determined by satisfying the following two inequalities

9 $$$$L\left({p}_{1N}\left|{\mu}_N/{\mu}_{0N}\right.\right)=\frac{\sum \limits_{i=0}^{c_1}\left(\begin{array}{*{20}l} n\\ {}i\end{array}\right){p_{1N}}^i{\left(1-{p}_{1N}\right)}^{n-i}}{\sum \limits_{i=0}^{c_1}\left(\begin{array}{*{20}l} n\\ {}i\end{array}\right){p_{1N}}^i{\left(1-{p}_{1N}\right)}^{n-i}+1-\sum \limits_{i=0}^{c_2}\left(\begin{array}{*{20}l} n\\ {}i\end{array}\right){p_{1N}}^i{\left(1-{p}_{1N}\right)}^{n-i}}\ge 1-\tilde{\alpha}$$

10 $$L\left({p}_{2N}\left|{\mu}_N/{\mu}_{0N}=1\right.\right)=\frac{\sum \limits_{i=0}^{c_1}\left(\begin{array}{*{20}l} n\\ {}i\end{array}\right){p_{2N}}^i{\left(1-{p}_{2N}\right)}^{n-i}}{\sum \limits_{i=0}^{c_1}\left(\begin{array}{*{20}l} n\\ {}i\end{array}\right){p_{2N}}^i{\left(1-{p}_{2N}\right)}^{n-i}+1-\sum \limits_{i=0}^{c_2}\left(\begin{array}{*{20}l} n\\ {}i\end{array}\right){p_{2N}}^i{\left(1-{p}_{2N}\right)}^{n-i}}\le \tilde{\beta}$$

where *p*_1*N*_ and *p*_2*N*_ are defined by

11 $${p}_{1N}=1-\left\{\exp \left(-{a}^{\beta }{\left(\mu /{\mu}_0\right)}^{-\beta }{\left(\Gamma \left(1/\beta \right)/\beta \right)}^{\beta }{\left(1+{I}_N\right)}^{\beta}\right)\left(1+{I}_N\right)\right\}+{I}_N$$

12 $${p}_{2N}=1-\left\{\exp \left(-{a}^{\beta }{\left(\Gamma \left(1/\beta \right)/\beta \right)}^{\beta }{\left(1+{I}_N\right)}^{\beta}\right)\left(1+{I}_N\right)\right\}+{I}_N$$

The estimated designed parameters of the proposed plan should be minimizing the average sample number (ASN) at an acceptable quality level. The ASN for the proposed plan with fraction defective (*p*) is derived to be

13 $$ASN=\frac{n}{P_a(p)+{P}_r(p)}$$

Therefore, the design parameters for the proposed plan with minimum sample size will be obtained by solving the below optimization technique

14 $${\displaystyle \begin{array}{*{20}l} \operatorname{Minimize}\ ASN\left({p}_{1N}\right)\\ {}\mathrm{subject}\ \mathrm{to}\ \\ {}L\left({p}_{1N}\right)\ge 1-\tilde{\alpha}\\ {}L\left({p}_{2N}\right)\le \tilde{\beta}\\ {}0\le {c}_1\le {c}_2\\ {}\mathrm{where}\kern0.5em n,{c}_1,{c}_2\in z\end{array}}$$

The values of the designed parameters *n, c*_*1*_ and *c*_*2*_ for various values of $\tilde{\beta }$ =0.25, 0.10, 0.05, 0.01; $\tilde{\alpha }=0.10$; *a* = 0.5 and 1.0, *μ*_*N*_/*μ*_0*N*_ =1.1, 1.2, 1.3, 1.4, 1.5, 1.8, 2.0 and *I*_*N*_ =0.0, 0.02, 0.04 and 0.05 when shape parameter *β* = 1, 2 and 3 are given in Tables 1, 2, 3, 4, 5 and 6. Tables 1 and 2 are shown for the exponential distribution case. For exponential distribution, it can be seen that the values of ASN decrease as the values of *a* increases from 0.5 to 1.0. On the other hand for other the same parameters, the values of *n* decreases as the values of *β* increases. Note here that the indeterminacy parameter *I*_*N*_ also plays a significant role in minimizing the sample size. As indeterminacy parameter *I*_*N*_ increases the ASN values are decreasing.

**Table 1:** The plan parameter when $\tilde{\alpha }=0.10;\beta =1$ and *a* = 0.50

$\tilde{\beta }$	$\frac{\mu_N}{\mu_{0N}}$	*I*_*U*_=0.00				*I*_*U*_=0.02				*I*_*U*_=0.04				*I*_*U*_=0.05			
		n	c_1_	c_2_	ASN	n	c_1_	c_2_	ASN	n	c_1_	c_2_	ASN	n	c_1_	c_2_	ASN
0.25	1.1	366	131	149	996.01	351	130	148	970.30	305	116	134	933.78	233	87	107	1123.61
0.25	1.2	180	64	70	262.79	144	52	59	244.14	163	62	68	241.62	116	43	51	236.76
0.25	1.3	67	21	27	135.08	71	24	29	121.47	57	19	25	124.91	81	30	34	117.39
0.25	1.4	61	20	23	83.75	47	15	19	79.12	31	9	14	78.47	33	10	15	79.74
0.25	1.5	46	14	17	65.19	26	7	11	56.28	25	7	11	55.12	31	10	13	49.83
0.25	1.8	30	9	10	34.14	24	7	9	33.65	18	5	7	26.81	27	9	10	31.03
0.25	2.0	24	7	8	27.96	21	6	7	24.49	13	3	5	20.91	20	6	7	23.32
0.10	1.1	–	–	–	–	–	–	–	–	–	–	–	–	–	–	–	–
0.10	1.2	195	65	77	388.69	233	83	93	365.45	220	81	91	349.86	185	68	79	341.30
0.10	1.3	114	36	44	197.37	102	33	41	184.31	104	35	43	181.35	89	30	38	171.55
0.10	1.4	74	22	28	121.14	81	26	31	115.70	69	22	28	113.54	65	21	27	110.40
0.10	1.5	64	19	23	87.09	42	11	17	86.67	29	7	13	83.12	46	14	19	79.02
0.10	1.8	32	8	11	44.72	31	8	11	43.09	30	8	11	41.58	16	3	7	38.95
0.10	2.0	23	5	8	35.50	26	6	9	36.49	22	5	8	32.58	18	4	7	30.19
0.05	1.1	–	–	–	–	–	–	–	–	–	–	–	–	–	–	–	–
0.05	1.2	292	99	112	454.10	254	88	102	440.72	230	82	96	417.79	250	92	105	406.48
0.05	1.3	154	49	58	231.40	117	37	47	217.69	113	37	47	211.21	114	38	48	206.78
0.05	1.4	78	22	30	144.10	91	28	35	137.49	91	29	36	133.57	89	29	36	133.17
0.05	1.5	55	14	21	103.05	44	11	18	97.99	61	18	24	93.72	57	17	23	90.86
0.05	1.8	30	6	11	53.55	40	10	14	53.72	35	9	13	50.20	24	5	10	49.66
0.05	2.0	29	6	10	44.08	30	7	10	38.81	24	5	9	38.60	32	8	11	39.32
0.01	1.1	–	–	–	–	–	–	–	–	–	–	–	–	–	–	–	–
0.01	1.2	393	130	150	586.50	371	127	147	565.50	357	127	146	530.22	343	124	143	520.89
0.01	1.3	191	58	72	290.60	196	62	76	283.30	178	58	72	271.54	187	63	76	262.20
0.01	1.4	141	41	51	185.60	111	32	43	174.80	116	35	46	170.80	108	33	44	168.20
0.01	1.5	91	24	33	131.10	91	25	34	127.60	76	21	30	117.90	89	26	35	123.50
0.01	1.8	50	11	17	67.53	45	10	16	62.85	39	8	15	62.79	46	11	17	61.25
0.01	2.0	40	8	13	51.86	30	5	11	49.49	32	6	12	49.98	37	8	13	46.90

Here hyphens (−) indicates that the parameters cannot be found to satisfy conditions

**Table 2:** The plan parameter when $\tilde{\alpha }=0.10;\beta =1$ and *a* = 1.0

$\tilde{\beta }$	$\frac{\mu_N}{\mu_{0N}}$	*I*_*U*_=0.00				*I*_*U*_=0.02				*I*_*U*_=0.04				*I*_*U*_=0.05			
		n	c_1_	c_2_	ASN	n	c_1_	c_2_	ASN	n	c_1_	c_2_	ASN	n	c_1_	c_2_	ASN
0.25	1.1	216	126	141	670.58	200	120	135	670.44	212	133	146	561.99	215	137	150	562.33
0.25	1.2	77	43	50	171.96	108	65	70	164.07	100	62	67	156.47	72	44	50	146.39
0.25	1.3	40	21	26	87.26	46	26	30	78.70	43	25	29	75.96	54	33	36	75.89
0.25	1.4	37	20	23	56.65	38	21	24	56.08	44	26	28	54.80	28	16	19	47.28
0.25	1.5	32	17	19	41.59	20	10	13	36.88	16	8	11	33.79	28	16	18	37.25
0.25	1.8	16	8	9	19.23	13	6	8	20.53	11	5	7	18.25	18	10	11	21.30
0.25	2.0	8	3	5	15.57	12	5	7	17.86	16	9	9	16.00	10	5	6	12.75
0.10	1.1	–	–	–	–	–	–	–	–	–	–	–	–	–	–	–	–
0.10	1.2	166	95	103	251.81	156	92	100	241.03	134	81	89	220.93	145	90	97	215.03
0.10	1.3	76	41	47	123.27	67	37	43	114.90	65	37	43	111.98	64	37	43	110.92
0.10	1.4	49	25	30	79.68	51	27	32	79.54	36	19	24	69.65	42	23	28	72.87
0.10	1.5	25	11	16	57.81	19	8	13	56.40	40	22	25	52.78	27	14	18	48.74
0.10	1.8	25	11	14	33.38	21	10	12	26.67	16	7	10	26.17	20	10	12	25.48
0.10	2.0	21	9	11	25.09	13	5	8	23.56	9	3	6	20.91	19	9	11	23.26
0.05	1.1	–	–	–	–	–	–	–	–	–	–	–	–	–	–	–	–
0.05	1.2	180	101	112	294.66	158	91	102	278.26	147	87	98	266.01	150	91	101	251.91
0.05	1.3	109	59	66	151.86	83	45	53	141.34	69	38	46	133.05	68	38	46	131.00
0.05	1.4	65	33	39	94.37	61	32	38	91.56	65	36	41	86.23	45	24	30	80.22
0.05	1.5	30	13	19	67.10	42	21	26	64.66	34	17	22	57.79	37	19	24	58.15
0.05	1.8	27	12	15	34.90	16	6	10	30.81	19	8	12	32.45	17	7	11	30.71
0.05	2.0	16	6	9	24.06	13	4	8	25.03	19	8	11	25.01	13	5	8	20.73
0.01	1.1	–	–	–	–	–	–	–	–	–	–	–	–	–	–	–	–
0.01	1.2	235	129	146	381.10	233	133	149	363.60	214	126	141	331.60	195	116	131	321.50
0.01	1.3	110	56	68	186.50	119	64	75	178.40	114	63	74	168.96	91	50	61	160.90
0.01	1.4	78	38	47	115.80	65	32	41	107.90	74	39	47	102.50	57	29	38	102.30
0.01	1.5	57	26	34	84.89	59	28	36	82.77	49	24	31	71.92	46	22	30	73.98
0.01	1.8	31	12	18	46.41	31	13	18	41.25	28	12	17	39.13	24	10	15	36.09
0.01	2.0	17	5	10	30.21	25	9	14	32.01	18	6	11	28.48	24	10	14	29.85

Here hyphens (−) indicates that the parameters cannot be found to satisfy conditions

**Table 3:** The plan parameter when $\tilde{\alpha }=0.10;\beta =2$ and *a* = 0.5

$\tilde{\beta }$	$\frac{\mu_N}{\mu_{0N}}$	*I*_*U*_=0.00				*I*_*U*_=0.02				*I*_*U*_=0.04				*I*_*U*_=0.05			
		n	c_1_	c_2_	ASN	n	c_1_	c_2_	ASN	n	c_1_	c_2_	ASN	n	c_1_	c_2_	ASN
0.25	1.1	256	38	47	517.20	284	46	54	491.58	293	51	58	456.12	319	58	64	449.42
0.25	1.2	98	13	17	155.09	71	9	14	150.09	62	8	13	136.88	60	8	13	134.22
0.25	1.3	53	6	9	85.43	51	6	9	80.86	48	6	9	76.77	26	2	6	74.76
0.25	1.4	38	4	6	54.61	37	4	6	52.16	28	3	5	42.59	33	4	6	47.73
0.25	1.5	25	2	4	40.34	31	3	5	45.48	33	4	5	38.75	22	2	4	35.43
0.25	1.8	28	3	3	28.00	16	1	2	20.75	15	1	2	19.54	14	1	2	18.53
0.25	2.0	21	2	2	21.00	21	2	2	21.00	20	2	2	20.00	8	0	1	11.94
0.10	1.1	462	69	81	778.83	425	67	79	731.36	345	56	69	693.87	421	73	84	666.03
0.10	1.2	149	19	25	231.97	113	14	21	218.82	127	18	24	204.39	92	12	19	197.78
0.10	1.3	72	7	12	126.10	77	9	13	114.70	73	9	13	108.58	71	9	13	105.81
0.10	1.4	62	6	9	83.19	58	6	9	78.82	48	5	8	68.56	37	3	7	69.39
0.10	1.5	42	3	6	61.36	31	2	5	52.13	29	2	5	49.61	25	1	5	58.24
0.10	1.8	23	1	3	34.22	22	1	3	32.40	18	0	3	35.08	20	1	3	29.81
0.10	2.0	15	0	2	26.36	14	0	2	24.93	15	0	2	23.66	13	0	2	22.99
0.05	1.1	486	70	86	921.90	559	88	102	860.99	518	86	100	809.80	453	76	91	789.07
0.05	1.2	173	21	29	276.86	157	20	28	258.41	142	19	27	243.33	145	20	28	237.39
0.05	1.3	93	9	15	148.49	105	12	17	142.08	69	7	13	127.94	90	11	16	126.23
0.05	1.4	56	4	9	97.89	45	3	8	89.79	58	5	10	90.71	59	6	10	82.73
0.05	1.5	46	3	7	74.60	36	2	6	64.99	42	3	7	65.87	33	2	6	60.20
0.05	1.8	44	3	5	51.50	19	0	3	37.07	18	0	3	35.08	17	0	3	34.70
0.05	2.0	28	1	3	35.44	26	1	3	33.32	24	1	3	31.33	23	1	3	30.38
0.01	1.1	–	–	–	–	–	–	–	–	–	–	–	–	–	–	–	–
0.01	1.2	206	23	35	351.10	195	23	35	331.10	219	29	40	312.40	207	28	39	301.03
0.01	1.3	135	13	21	187.90	113	11	19	169.80	113	12	20	166.28	97	10	18	154.38
0.01	1.4	88	7	13	120.60	84	7	13	113.10	79	7	13	107.50	77	7	13	104.49
0.01	1.5	64	4	9	87.32	60	4	9	82.88	54	3	9	82.24	56	4	9	75.69
0.01	1.8	27	0	4	49.47	38	1	5	50.64	35	1	5	48.14	39	2	5	46.16
0.01	2.0	40	1	4	46.08	35	1	4	42.60	32	1	4	40.05	22	0	3	31.76

Here hyphens (−) indicates that the parameters cannot be found to satisfy conditions

**Table 4:** The plan parameter when $\tilde{\alpha }=0.10;\beta =2$ and *a* = 1.00

$\tilde{\beta }$	$\frac{\mu_N}{\mu_{0N}}$	*I*_*U*_=0.00				*I*_*U*_=0.02				*I*_*U*_=0.04				*I*_*U*_=0.05			
		n	c_1_	c_2_	ASN	n	c_1_	c_2_	ASN	n	c_1_	c_2_	ASN	n	c_1_	c_2_	ASN
0.255	1.1	139	70	74	180.96	98	50	56	167.43	105	57	62	158.45	83	45	51	152.44
0.25	1.2	27	11	15	55.27	39	19	21	49.78	31	15	18	48.58	30	15	18	48.38
0.25	1.3	17	6	9	31.20	19	8	10	26.98	18	8	10	26.05	19	9	11	27.77
0.25	1.4	17	7	8	20.21	11	4	6	18.62	11	4	6	17.60	18	9	9	18.00
0.25	1.5	11	4	5	13.71	19	9	8	16.85	8	3	4	10.47	5	1	3	11.57
0.25	1.8	5	1	2	6.95	8	3	3	8.00	3	0	2	10.82	6	2	3	8.23
0.25	2.0	6	1	2	7.42	5	1	2	6.75	6	2	2	6.00	9	4	3	7.76
0.10	1.1	138	65	75	263.63	169	86	94	250.98	134	70	79	233.82	137	74	82	220.09
0.10	1.2	57	25	29	78.21	41	17	23	79.30	45	21	26	75.79	39	18	23	67.40
0.10	1.3	25	9	13	43.98	25	10	13	36.43	24	10	13	34.72	30	14	16	36.26
0.10	1.4	16	5	8	26.78	15	5	8	26.29	22	9	11	26.89	12	4	7	23.41
0.10	1.5	10	2	5	19.27	15	5	7	19.59	11	3	6	19.95	11	3	6	18.92
0.10	1.8	9	2	4	13.62	4	0	2	8.74	10	3	4	11.31	10	3	4	11.16
0.10	2.0	9	2	3	10.25	4	0	2	8.74	8	1	3	9.66	8	2	3	9.05
0.05	1.1	189	89	101	313.09	181	90	101	289.79	181	95	105	271.36	170	91	101	261.00
0.05	1.2	56	23	29	88.23	59	26	32	91.16	49	22	28	80.65	46	21	27	78.19
0.05	1.3	37	14	18	50.18	31	12	16	44.56	32	13	17	43.38	25	10	14	38.63
0.05	1.4	26	9	12	33.51	17	5	9	30.35	12	3	7	27.18	21	8	11	28.07
0.05	1.5	19	6	8	22.62	12	3	6	19.74	14	4	7	20.04	11	3	6	18.92
0.05	1.8	10	2	4	13.24	10	2	4	12.52	8	1	4	13.51	9	2	4	11.65
0.05	2.0	7	0	3	11.67	9	2	3	9.99	8	1	3	9.66	9	2	4	11.65
0.01	1.1	272	127	144	405.20	255	125	141	370.60	229	117	133	349.70	184	94	111	345.60
0.01	1.2	78	31	40	115.10	72	30	39	111.20	70	31	39	98.57	56	24	33	98.24
0.01	1.3	35	11	18	60.36	39	14	20	54.33	37	14	20	52.56	32	12	18	49.45
0.01	1.4	33	10	15	40.78	29	9	14	37.17	25	8	13	34.68	27	9	14	34.57
0.01	1.5	19	4	9	29.01	28	9	12	30.69	18	5	9	24.68	17	4	9	25.42
0.01	1.8	13	2	5	15.97	9	1	4	13.37	12	2	5	14.35	11	2	5	14.17
0.01	2.0	15	3	5	16.25	12	2	4	13.07	6	0	3	11.13	6	0	3	10.62

**Table 5:** The plan parameter when $\tilde{\alpha }=0.10;\beta =3$ and *a* = 0.5

$\tilde{\beta }$	$\frac{\mu_N}{\mu_{0N}}$	*I*_*U*_=0.00				*I*_*U*_=0.02				*I*_*U*_=0.04				*I*_*U*_=0.05			
		n	c_1_	c_2_	ASN	n	c_1_	c_2_	ASN	n	c_1_	c_2_	ASN	n	c_1_	c_2_	ASN
0.25	1.1	292	19	25	517.61	245	17	23	458.13	277	22	27	429.66	267	22	27	414.40
0.25	1.2	85	4	7	146.81	91	5	8	149.65	73	4	7	126.55	70	4	7	122.04
0.25	1.3	30	0	3	94.07	51	2	4	78.45	46	2	4	72.19	44	2	4	69.43
0.25	1.4	25	0	2	53.46	46	2	3	56.55	42	2	3	51.90	21	0	2	44.60
0.25	1.5	35	1	2	45.38	35	1	2	44.23	30	1	2	38.96	30	1	2	38.48
0.25	1.8	20	0	1	29.15	21	0	1	29.19	17	0	1	24.90	27	1	1	27.00
0.25	2.0	20	0	1	29.15	20	0	1	28.34	18	0	1	25.81	17	0	1	24.57
0.10	1.1	403	25	34	759.91	443	31	39	701.00	399	30	38	642.41	385	30	38	617.97
0.10	1.2	123	5	10	235.19	149	8	12	218.34	138	8	12	202.77	133	8	12	195.45
0.10	1.3	111	5	7	135.49	55	1	5	128.82	74	3	6	111.25	50	1	5	113.77
0.10	1.4	38	0	3	86.02	34	0	3	80.99	58	2	4	76.59	56	2	4	73.83
0.10	1.5	63	2	3	71.39	47	1	3	67.15	45	1	3	62.66	41	1	3	59.74
0.10	1.8	47	1	2	54.83	43	1	2	50.37	41	1	2	47.54	27	0	2	45.89
0.10	2.0	32	0	1	38.68	31	0	1	36.81	28	0	1	33.57	24	0	1	30.13
0.05	1.1	494	30	41	890.71	470	31	42	834.52	412	29	40	764.15	432	32	43	751.53
0.05	1.2	190	9	14	274.71	139	6	12	257.00	128	6	12	241.07	157	9	14	227.78
0.05	1.3	100	3	7	153.78	110	4	8	154.20	69	2	6	123.22	82	3	7	127.89
0.05	1.4	61	1	4	96.63	56	1	4	89.64	52	1	4	83.11	70	2	5	92.28
0.05	1.5	44	0	3	81.11	43	0	3	73.85	37	0	3	70.19	38	0	3	66.19
0.05	1.8	41	0	2	57.27	36	0	2	52.46	34	0	2	48.78	33	0	2	47.05
0.05	2.0	36	0	1	41.67	34	0	1	39.07	32	0	1	36.56	30	0	1	34.59
0.01	1.1	–	–	–	–	–	–	–	–	–	–	–	–	–	–	–	–
0.01	1.2	241	10	18	356.05	223	10	18	330.00	222	11	19	312.30	185	9	17	289.40
0.01	1.3	155	5	10	197.70	116	3	9	182.40	133	5	10	169.60	104	3	9	162.40
0.01	1.4	100	2	6	130.80	92	2	6	121.10	74	1	6	120.80	82	2	6	108.20
0.01	1.5	61	0	4	100.60	56	0	4	93.47	52	0	4	86.59	50	0	4	83.51
0.01	1.8	54	0	2	62.97	53	0	3	72.01	51	0	3	66.94	46	0	3	64.22
0.01	2.0	54	0	2	62.97	52	0	2	59.44	49	0	2	55.56	45	0	2	52.19

Here hyphens (−) indicates that the parameters cannot be found to satisfy conditions

**Table 6:** The plan parameter when $\tilde{\alpha }=0.10;\beta =3$ and *a* = 1.0

$\tilde{\beta }$	$\frac{\mu_N}{\mu_{0N}}$	*I*_*U*_=0.00				*I*_*U*_=0.02				*I*_*U*_=0.04				*I*_*U*_=0.05			
		n	c_1_	c_2_	ASN	n	c_1_	c_2_	ASN	n	c_1_	c_2_	ASN	n	c_1_	c_2_	ASN
0.25	1.1	78	32	39	136.44	65	31	34	86.96	66	34	36	79.07	46	23	27	77.00
0.25	1.2	25	8	12	42.95	23	10	11	26.67	17	7	9	24.71	15	6	9	31.15
0.25	1.3	19	6	8	24.45	11	4	5	13.76	10	3	5	15.57	5	1	3	12.06
0.25	1.4	13	3	5	16.83	9	2	4	13.73	10	4	4	10.00	8	2	4	12.77
0.25	1.5	11	2	4	14.20	9	3	3	9.00	8	3	3	8.00	7	2	3	8.79
0.25	1.8	4	0	1	5.27	3	0	1	4.56	4	1	1	4.00	4	1	1	4.00
0.25	2.0	4	0	1	5.27	3	0	1	4.56	4	1	1	4.00	4	1	1	4.00
0.10	1.1	44	18	23	89.62	59	25	32	120.98	68	32	38	112.30	64	31	37	109.60
0.10	1.2	16	5	8	30.57	27	10	13	37.35	20	7	11	37.37	15	5	9	36.41
0.10	1.3	11	3	5	17.54	15	4	7	22.85	17	6	8	21.59	9	2	5	18.53
0.10	1.4	10	3	4	12.46	13	4	5	14.73	9	2	4	12.65	14	5	6	15.58
0.10	1.5	9	2	3	10.65	7	1	3	11.14	8	2	3	9.41	4	0	2	8.24
0.10	1.8	3	0	1	4.75	8	2	2	8.00	6	1	2	7.17	3	0	1	4.27
0.10	2.0	3	0	1	4.75	7	0	2	8.34	4	0	1	4.87	4	0	1	4.78
0.05	1.1	98	40	48	152.82	95	42	49	138.53	69	31	39	129.53	81	39	46	125.46
0.05	1.2	22	6	11	46.59	28	9	14	45.58	27	10	14	39.85	19	6	11	40.85
0.05	1.3	16	4	7	24.25	18	5	8	24.21	14	4	7	21.72	14	4	7	20.45
0.05	1.4	17	4	6	19.47	15	4	6	18.10	8	1	4	14.70	10	2	5	16.09
0.05	1.5	8	1	3	11.62	12	3	4	13.10	11	3	4	12.11	7	1	3	9.84
0.05	1.8	6	0	2	8.73	7	1	2	8.00	6	0	2	7.62	5	0	2	7.40
0.05	2.0	5	0	1	5.87	6	0	2	8.12	7	1	2	7.76	4	0	1	4.78
0.01	1.1	136	54	66	197.50	113	47	59	180.40	107	48	59	165.30	104	48	59	160.40
0.01	1.2	33	9	16	60.20	37	12	18	52.96	34	11	18	53.17	36	13	19	49.51
0.01	1.3	23	5	10	32.96	16	3	8	29.75	24	7	11	28.94	17	4	9	27.88
0.01	1.4	11	1	5	20.74	18	4	7	20.91	17	4	7	19.56	14	2	7	21.09
0.01	1.5	12	1	5	18.73	10	1	4	13.81	13	2	5	15.03	9	1	4	12.57
0.01	1.8	7	0	2	8.80	10	1	3	11.21	6	0	2	7.62	8	0	3	9.87
0.01	2.0	8	0	2	9.19	8	0	2	8.84	8	0	2	8.58	7	0	2	7.82

## Results

A comparative study is carried out between the proposed sampling plans with the existing sampling plans available in the literature with respect to the sample size in this section. We know the cost of the study is always directly proportional to the sample size, a plan is said to be economical if it requires a smaller number of samples for testing the hypothesis about the daily new deaths from COVID-19. The proposed repetitive sampling plan under uncertainty/indeterminacy for Weibull distribution is the generalization of the testing average wind speed using sampling plan for Weibull distribution under indeterminacy plan developed by [42]. The comparison for the proposed and the existing sampling plan for Weibull distribution under indeterminacy plan developed by [42] are displayed in Tables 7 and 8 for $\tilde{\alpha }=0.10;\beta =2$ at *a* = 0.5 and 1.0. The developed sampling plan reduces to the existing sampling plan when *c*_*1*_ *= c*_*2*_ *= c*. From Tables 7 and 8, it is noticed that the values of the sample size required for testing *H*_0_ : *μ*_*N*_ = *μ*_0*N*_ smaller for the proposed sampling plan as compared with the existing sampling plan developed by [42]. For example, when *μ*_*N*_/*μ*_0*N*_ =1.1 and *a* =0.5 from Table 7, it can be seen that ASN = 491.58 from the plan proposed sampling plan whereas existing sampling plan sample size *n* = 617 when *I*_*N*_ =0.02, *β* = 2 and *a* = 0.5. Hence, the proposed sampling plan is more economical than the existing sampling plan.

**Table 7:** Sample size comparison between the proposed plan and existing plan for $\tilde{\alpha }=0.10;\beta =2$ and *a* = 0.5

$\tilde{\beta }$	$\frac{\mu_N}{\mu_{0N}}$	*I*_*U*_=0.00		*I*_*U*_=0.02		*I*_*U*_=0.04		*I*_*U*_=0.05	
		Proposed	Existing	Proposed	Existing	Proposed	Existing	Proposed	Existing
0.25	1.1	517.20	646	491.58	617	456.12	573	449.42	558
0.25	1.2	155.09	198	150.09	181	136.88	172	134.22	167
0.25	1.3	85.43	110	80.86	103	76.77	97	74.76	94
0.25	1.4	54.61	66	52.16	62	42.59	59	47.73	58
0.25	1.5	40.34	47	45.48	45	38.75	42	35.43	41
0.25	1.8	28.00	29	20.75	27	19.54	25	18.53	25
0.25	2.0	21.00	21	21.00	20	20.00	19	11.94	19
0.10	1.1	778.83	1122	731.36	1049	693.87	993	666.03	967
0.10	1.2	231.97	327	218.82	315	204.39	298	197.78	285
0.10	1.3	126.10	174	114.70	164	108.58	155	105.81	151
0.10	1.4	83.19	117	78.82	110	68.56	105	69.39	101
0.10	1.5	61.36	84	52.13	79	49.61	76	58.24	73
0.10	1.8	34.22	50	32.40	47	35.08	45	29.81	44
0.10	2.0	26.36	36	24.93	34	23.66	32	22.99	31
0.05	1.1	921.90	1467	860.99	1370	809.80	1297	789.07	1257
0.05	1.2	276.86	435	258.41	411	243.33	383	237.39	373
0.05	1.3	148.49	230	142.08	218	127.94	213	126.23	200
0.05	1.4	97.89	153	89.79	145	90.71	142	82.73	132
0.05	1.5	74.60	112	64.99	106	65.87	100	60.20	98
0.05	1.8	51.50	64	37.07	60	35.08	57	34.70	55
0.05	2.0	35.44	49	33.32	46	31.33	44	30.38	44
0.01	1.1	–	646	–	617	–	573	–	558
0.01	1.2	351.10	198	331.10	181	312.40	172	301.03	167
0.01	1.3	187.90	110	169.80	103	166.28	97	154.38	94
0.01	1.4	120.60	66	113.10	62	107.50	59	104.49	58
0.01	1.5	87.32	47	82.88	45	82.24	42	75.69	41
0.01	1.8	49.47	29	50.64	27	48.14	25	46.16	25
0.01	2.0	46.08	21	42.60	20	40.05	19	31.76	19

Here hyphens (−) indicates that the parameters cannot be found to satisfy conditions

**Table 8:** Sample size comparison between the proposed plan and existing plan for $\tilde{\alpha }=0.10;\beta =2$ and *a* = 1.0

$\tilde{\beta }$	$\frac{\mu_N}{\mu_{0N}}$	*I*_*U*_=0.00		*I*_*U*_=0.02		*I*_*U*_=0.04		*I*_*U*_=0.05	
		Proposed	Existing	Proposed	Existing	Proposed	Existing	Proposed	Existing
0.25	1.1	180.96	229	167.43	206	158.45	190	152.44	188
0.25	1.2	55.27	65	49.78	60	48.58	59	48.38	56
0.25	1.3	31.20	36	26.98	35	26.05	33	27.77	32
0.25	1.4	20.21	25	18.62	23	17.60	22	18.00	18
0.25	1.5	13.71	17	16.85	16	10.47	15	11.57	15
0.25	1.8	6.95	11	8.00	11	10.82	10	8.23	9
0.25	2.0	7.42	10	6.75	10	6.00	9	7.76	9
0.10	1.1	263.63	371	250.98	352	233.82	324	220.09	317
0.10	1.2	78.21	109	79.30	104	75.79	99	67.40	97
0.10	1.3	43.98	56	36.43	55	34.72	51	36.26	51
0.10	1.4	26.78	38	26.29	36	26.89	35	23.41	35
0.10	1.5	19.27	30	19.59	28	19.95	27	18.92	24
0.10	1.8	13.62	15	8.74	14	11.31	14	11.16	13
0.10	2.0	10.25	13	8.74	13	9.66	12	9.05	9
0.05	1.1	313.09	494	289.79	462	271.36	431	261.00	416
0.05	1.2	88.23	146	91.16	138	80.65	129	78.19	119
0.05	1.3	50.18	73	44.56	69	43.38	66	38.63	65
0.05	1.4	33.51	49	30.35	44	27.18	42	28.07	41
0.05	1.5	22.62	38	19.74	37	20.04	35	18.92	30
0.05	1.8	13.24	22	12.52	21	13.51	20	11.65	18
0.05	2.0	11.67	20	9.99	18	9.66	17	11.65	16
0.01	1.1	405.20	229	370.60	206	349.70	190	345.60	188
0.01	1.2	115.10	65	111.20	60	98.57	59	98.24	56
0.01	1.3	60.36	36	54.33	35	52.56	33	49.45	32
0.01	1.4	40.78	25	37.17	23	34.68	22	34.57	18
0.01	1.5	29.01	17	30.69	16	24.68	15	25.42	15
0.01	1.8	15.97	11	13.37	11	14.35	10	14.17	9
0.01	2.0	16.25	10	13.07	10	11.13	9	10.62	9

## Discussions

**Fig. 1:**
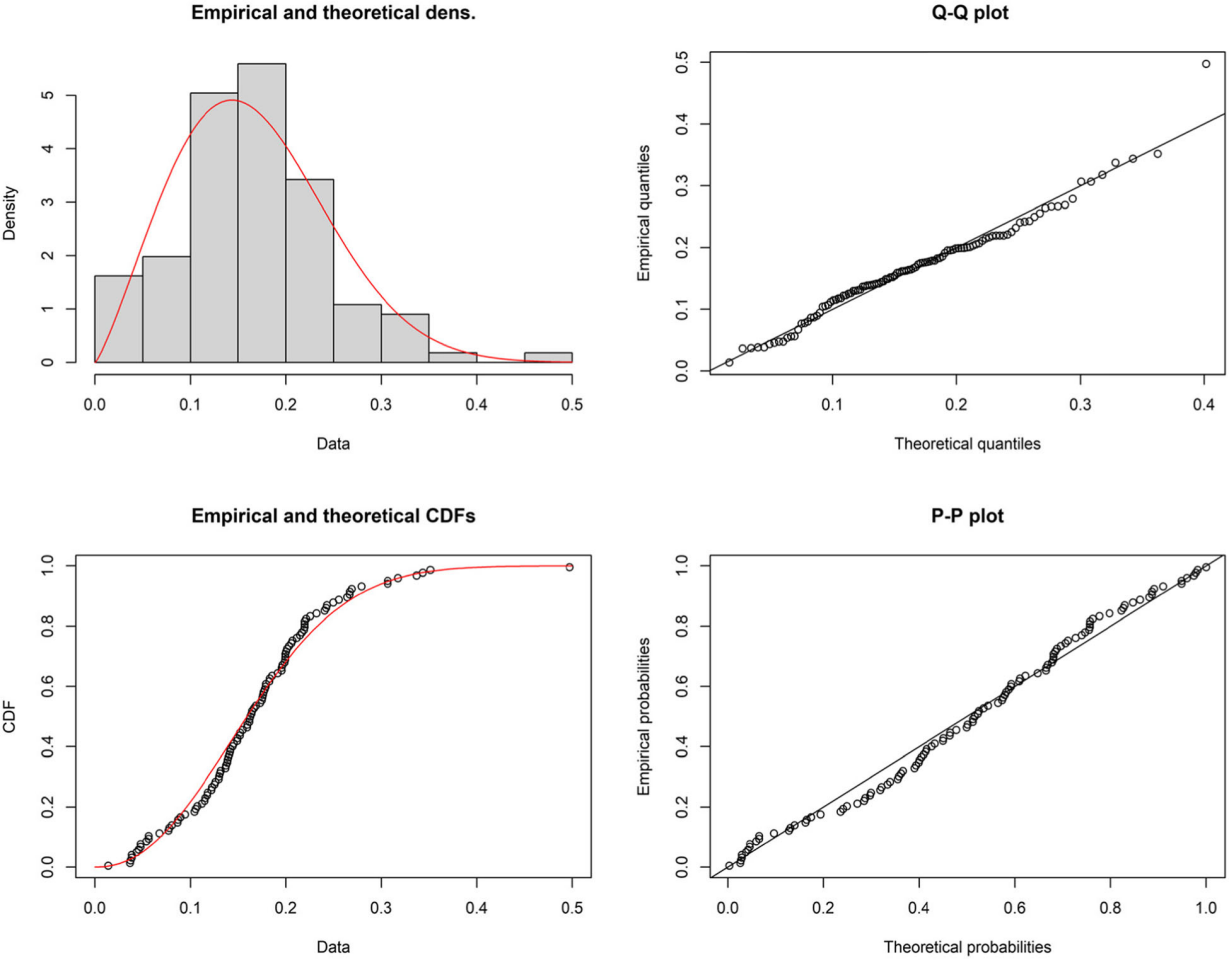
The empirical and theoretical pdfs, empirical and theoretical cdfs, Q-Q plots and p-p plot for the WD for the daily new deaths

At this juncture, application of the proposed methodology will be illustrated using COVID-19 data belong to Italy of 111 days that are recorded from 1 April to 20 July 2020. The data are available at https://covid19.who.int/. This data is made up of the ratio of daily new deaths (i.e. daily number of deaths over new cases). The data is reported in Table 9. We have taken this data from [46] and they studied applications of COVID-19 data for Kumaraswamy inverted Topp-Leone distribution. Coronavirus disease (COVID-19) is an infectious disease caused by a newly discovered coronavirus. A large number of people affected by the COVID-19 virus and it are infected at random and uncertain, the COVID-19 data follows a certain statistical distribution under neutrosophic statistics. The World health organization and different countries’ health administrators are involved to check the daily affected cases, recovered cases and deaths under indeterminacy. It is found that the COVID-19 data follows the Weibull distribution with shape parameter $\hat{\beta}=$ 2.2222 with the standard error (SE) as 0.1596 and scale parameter $\hat{\alpha}=0.1880$ with SE value as 0.00845. The Kolmogorov-Smirnov test and it *p* value are D = 0.0684 and *p* = 0.6766. The goodness of fit of the Weibull distribution is highlight by depicts the histogram and quantile-quantile (Q-Q) plot in Fig. 1. We also applied various life distributions to fit the COVID-19 data set for the intention of comparative study. We have considered here the existing three models like odds Weibull distribution (OWD), Nadarajah-Haghighi distribution (NHD) and Exponentiated Nadarajah-Haghighi distribution (ENHD) for the same data. For more details please refer to [47].

**Table 9:** COVID-19 data belong to Italy from 1 April to 20 July 2020

0.0138	0.0365	0.0372	0.0385	0.0385	0.0435	0.0457	0.0476	0.0476	0.0537
0.0561	0.0562	0.0673	0.0769	0.0777	0.0802	0.0864	0.0870	0.0894	0.0942
0.1041	0.1053	0.1071	0.1119	0.1149	0.1154	0.1176	0.1180	0.1221	0.1227
0.1253	0.1264	0.1297	0.1302	0.1311	0.1319	0.1369	0.1375	0.1387	0.1390
0.1398	0.1408	0.1417	0.1421	0.1443	0.1456	0.1491	0.1493	0.1520	0.1522
0.1548	0.1593	0.1597	0.1619	0.1620	0.1628	0.1641	0.1646	0.1666	0.1686
0.1730	0.1749	0.1754	0.1761	0.1767	0.1779	0.1789	0.1791	0.1827	0.1831
0.1856	0.1915	0.1956	0.1957	0.1965	0.1987	0.1993	0.1994	0.1994	0.2003
0.2012	0.2032	0.2057	0.2070	0.2113	0.2148	0.2167	0.2190	0.2195	0.2195
0.2196	0.2212	0.2254	0.2321	0.2406	0.2421	0.2430	0.2495	0.2555	0.2641
0.2667	0.2668	0.2690	0.2792	0.3067	0.3067	0.3176	0.3371	0.3436	0.3515
0.4972

Pdf and cdf of Weibull distribution are respectively

$$f(x)=\left(\frac{\beta }{\alpha}\right){\left(\frac{x}{\alpha}\right)}^{\beta -1}{e}^{-{\left(\frac{x}{\alpha}\right)}^{\beta }};x>0,\alpha >0,\beta >0$$

and $$F(x)=1-{e}^{-{\left(\frac{x}{\alpha}\right)}^{\beta }}$$; *x* > 0, *α* > 0, *β* > 0

Pdf and cdf of odds Weibull distribution (OWD) are respectively (suggested by [48])

$$f(x)=\left(\frac{\alpha \beta}{x}\right){\left(\frac{x}{\theta}\right)}^{\alpha }{e}^{{\left(\frac{x}{\theta}\right)}^{\alpha }}{\left({e}^{{\left(\frac{x}{\theta}\right)}^{\alpha }}-1\right)}^{\beta -1}{\left[1+{\left({e}^{{\left(\frac{x}{\theta}\right)}^{\alpha }}-1\right)}^{\beta}\right]}^{-2};x>0,\alpha <0,0<\left(\beta, \theta \right)$$

and $$F(x)=1-{\left[1+{\left({e}^{{\left(\frac{x}{\theta}\right)}^{\alpha }}-1\right)}^{\beta}\right]}^{-1}$$; *x* > 0, *α* < 0, 0 < (*β*, *θ*).

Pdf and cdf of Nadarajah-Haghighi distribution (NHD) are respectively (see [49])

$$f(x)=\left(\alpha \lambda \right){\left(1+\lambda x\right)}^{\alpha -1}{e}^{1-{\left(1+\lambda x\right)}^{\alpha }};x>0,\alpha >0,\lambda >0$$

and $$F(x)=1-{e}^{1-{\left(1+\lambda x\right)}^{\alpha }}$$; *x* > 0, *α* > 0, *λ* > 0.

Pdf and cdf of Exponentiated Nadarajah-Haghighi distribution (ENHD) are respectively (see [49])

$$f(x)=\left(\alpha \lambda \theta \right){\left(1+\lambda x\right)}^{\alpha -1}{e}^{1-{\left(1+\lambda x\right)}^{\alpha }}{\left(1-{e}^{1-{\left(1+\lambda x\right)}^{\alpha }}\right)}^{\theta -1};x>0,\alpha >0,\lambda >0,\theta >0$$

and $$F(x)={\left(1-{e}^{1-{\left(1+\lambda x\right)}^{\alpha }}\right)}^{\theta }$$; *x* > 0, *α* > 0, *λ* > 0, *θ* > 0.

**Fig. 2:**
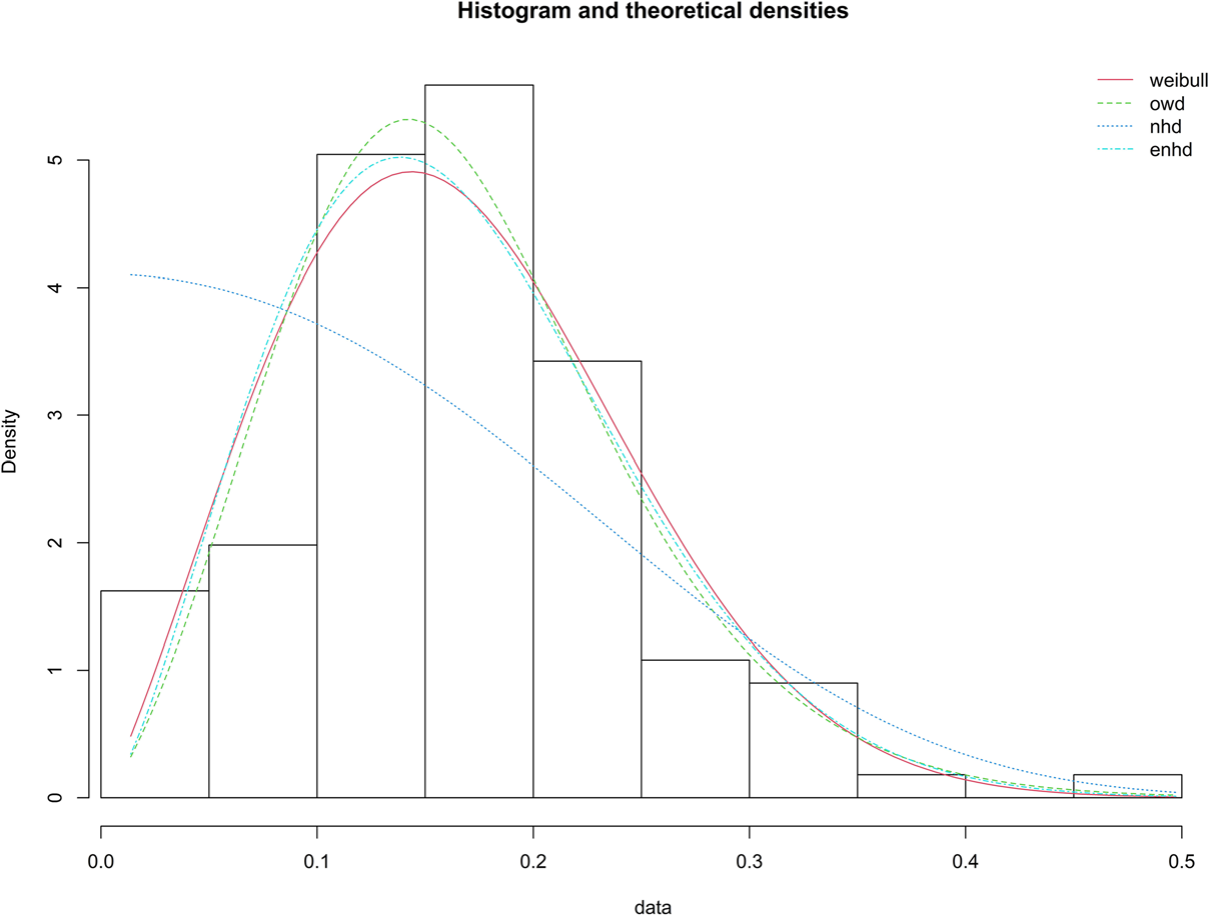
Comparison of various models for the daily new deaths

We have estimated the parameters and good fit for the COVID-19 data for WD, OWD, NHD and ENHD, and are reported in Table 10 and depicted in Fig. 2. From Table 10 and Fig. 2 it is noticed that WD shows less AIC, BIC and -2logLL, moreover OWD and NHD are not fitted for COVID-19 data. Hence, Weibull distribution shows a good fit for the COVID-19 data belongs to Italy. The plan parameters for this shape parameter are shown in Tables 11 and 12. For the proposed plan, the shape parameter is ${\hat{\beta}}_N=\left(1+0.04\right)\times 2.2222\approx 2.31$ when *I*_*U*_ =0.04.

**Table 10:** Estimation and Goodness of fit measures of fitted distribution for daily new deaths

Dist	MLEs of the parameters			KS test *p*-value	-2*logLL*	AIC	BIC
WD	$\hat{\alpha}$ =0.1880	$\hat{\beta}$ =2.2222	–	0.6766	−257.1131	−253.1131	−247.6940
OWD	$\hat{\alpha}$=1.7988	$\hat{\beta}$ =1.3225	$\hat{\theta}$ =0.1943	2.2e-16	−258.5084	−252.5084	−244.3798
NHD	$\hat{\alpha}$=116.5132	$\hat{\lambda}$=0.0353		8.549e-06	−221.1094	−217.1095	−211.6904
ENHD	$\hat{\alpha}$=3.7626	$\hat{\lambda}$=1.6968	$\hat{\theta}$=2.5732	0.6324	−256.9344	−250.9344	−242.8058

**Table 11:** The plan parameter when $\tilde{\alpha }=0.10;\beta =2.2222$ and *a* = 0.5

$\tilde{\beta }$	$\frac{\mu_N}{\mu_{0N}}$	*I*_*U*_=0.00				*I*_*U*_=0.02				*I*_*U*_=0.04				*I*_*U*_=0.05			
		n	c_1_	c_2_	ASN	n	c_1_	c_2_	ASN	n	c_1_	c_2_	ASN	n	c_1_	c_2_	ASN
0.25	1.1	312	40	47	507.20	287	39	46	471.79	305	45	51	447.47	256	38	45	428.73
0.25	1.2	87	9	13	150.31	106	13	16	147.51	100	13	16	139.26	52	5	10	135.80
0.25	1.3	60	6	8	80.77	57	6	8	76.36	60	7	9	79.08	41	4	7	72.91
0.25	1.4	26	1	4	59.83	36	3	5	53.13	46	5	6	52.93	34	3	5	49.13
0.25	1.5	37	3	4	43.78	21	1	3	36.92	31	3	4	37.42	19	1	3	33.81
0.25	1.8	25	2	2	25.00	21	1	2	26.09	19	1	2	23.99	22	2	2	22.00
0.25	2.0	27	2	2	27.00	20	1	2	25.33	10	0	1	14.66	11	0	1	15.30
0.10	1.1	431	53	64	742.14	413	54	65	703.25	376	52	63	655.88	406	59	69	635.70
0.10	1.2	140	14	20	232.64	143	16	21	208.70	110	12	18	195.90	114	13	19	195.07
0.10	1.3	74	6	10	117.74	70	6	10	110.54	74	7	11	110.43	82	9	12	106.44
0.10	1.4	47	3	6	73.54	44	3	6	69.36	51	4	7	72.13	48	4	7	70.57
0.10	1.5	42	2	5	63.55	36	2	5	61.50	26	1	4	50.99	36	2	5	54.79
0.10	1.8	18	0	2	31.17	26	1	3	38.16	16	0	2	27.66	25	1	3	35.16
0.10	2.0	19	0	2	31.25	17	0	2	29.34	23	1	2	26.93	23	1	2	26.63
0.05	1.1	507	61	75	883.90	544	71	84	836.31	486	67	80	773.35	472	67	80	752.06
0.05	1.2	172	17	24	266.94	154	16	23	246.46	152	17	24	238.68	141	16	23	225.79
0.05	1.3	99	8	13	146.61	94	8	13	136.91	80	7	12	124.05	78	7	12	120.03
0.05	1.4	54	3	7	88.62	61	4	8	89.14	56	4	8	85.25	55	4	8	82.24
0.05	1.5	53	3	6	71.76	51	3	6	67.35	46	3	6	63.79	32	1	5	62.18
0.05	1.8	33	1	3	41.98	30	1	3	39.13	29	1	3	37.07	27	1	3	35.64
0.05	2.0	23	0	2	31.96	21	0	2	29.89	19	0	2	27.97	20	0	2	27.50
0.01	1.1	–	–	–	–	–	–	–	–	–	–	–	–	–	–	–	–
0.01	1.2	204	18	29	341.70	192	18	29	321.80	207	22	32	298.90	216	24	34	297.80
0.01	1.3	133	10	17	181.10	93	6	14	172.80	109	9	16	156.30	106	9	16	151.50
0.01	1.4	87	5	11	127.20	84	5	11	117.10	69	4	10	106.60	58	3	9	99.95
0.01	1.5	56	2	7	88.85	63	3	8	89.39	51	2	7	77.16	49	2	7	75.48
0.01	1.8	43	1	4	53.38	47	1	5	59.58	39	1	4	47.41	37	1	4	45.83
0.01	2.0	31	0	3	43.35	30	0	3	40.74	27	0	3	38.37	28	0	3	37.24

Here hyphens (−) indicates that the parameters cannot be found to satisfy conditions

**Table 12:** The plan parameter when $\tilde{\alpha }=0.10;\beta =2.2222$ and *a* = 1.0

$\tilde{\beta }$	$\frac{\mu_N}{\mu_{0N}}$	*I*_*U*_=0.00				*I*_*U*_=0.02				*I*_*U*_=0.04				*I*_*U*_=0.05			
		n	c_1_	c_2_	ASN	n	c_1_	c_2_	ASN	n	c_1_	c_2_	ASN	n	c_1_	c_2_	ASN
0.25	1.1	113	55	59	151.82	86	43	48	136.48	80	42	47	130.72	73	39	44	122.99
0.25	1.2	38	17	19	48.43	41	20	21	45.66	24	11	14	41.34	20	9	12	36.62
0.25	1.3	14	5	7	21.80	15	6	8	23.22	21	10	11	24.55	14	6	8	21.94
0.25	1.4	16	6	7	18.75	14	5	7	20.53	12	5	6	14.76	12	5	6	14.55
0.25	1.5	9	3	4	11.62	11	4	5	13.48	5	1	3	12.11	6	2	3	8.28
0.25	1.8	8	2	3	9.85	4	0	2	8.97	5	1	2	6.60	6	2	3	8.28
0.25	2.0	3	0	1	4.60	6	2	2	6.00	6	1	2	7.05	5	1	2	6.48
0.10	1.1	144	67	75	221.16	109	52	61	209.33	114	58	66	191.67	104	54	62	184.96
0.10	1.2	44	18	22	64.25	44	19	23	62.91	38	17	21	56.83	35	16	20	54.71
0.10	1.3	23	8	11	33.52	27	11	13	33.00	18	6	10	32.88	16	6	9	27.15
0.10	1.4	18	6	8	23.33	17	6	8	22.23	11	3	6	20.57	16	6	8	20.58
0.10	1.5	8	1	4	17.30	14	4	6	17.44	8	2	4	12.83	12	4	6	16.54
0.10	1.8	9	2	3	10.36	4	0	2	8.97	10	3	4	11.40	8	2	3	9.12
0.10	2.0	5	0	2	8.54	5	0	2	7.95	6	1	2	7.05	5	0	2	7.19
0.05	1.1	178	82	92	262.01	168	82	91	240.57	126	63	73	220.31	123	63	73	217.07
0.05	1.2	45	17	23	76.34	50	21	26	71.19	47	20	26	72.09	30	12	18	65.41
0.05	1.3	33	12	15	40.86	26	9	13	37.70	18	6	10	32.88	24	9	13	35.44
0.05	1.4	14	3	7	27.30	16	4	8	26.27	16	5	8	22.68	18	6	9	23.65
0.05	1.5	13	3	6	20.78	10	2	5	18.00	12	3	6	18.41	12	3	6	17.53
0.05	1.8	10	2	4	13.55	8	1	3	10.32	11	3	4	11.95	11	3	4	11.81
0.05	2.0	8	1	3	10.95	7	1	3	10.57	9	2	3	9.82	6	1	2	6.95
0.01	1.1	195	86	102	325.80	200	94	109	305.97	214	107	121	292.44	185	94	108	275.80
0.01	1.2	69	26	34	95.71	59	23	31	88.26	54	22	30	83.99	63	27	35	85.42
0.01	1.3	38	12	18	52.35	29	9	15	46.76	32	11	17	46.48	27	9	15	43.28
0.01	1.4	21	5	10	33.36	20	5	10	31.56	19	5	10	30.11	19	5	10	28.11
0.01	1.5	21	4	9	27.10	14	2	7	24.13	16	4	8	23.27	14	3	7	19.98
0.01	1.8	16	3	5	16.99	10	1	4	13.08	11	2	4	12.32	9	1	4	12.15
0.01	2.0	10	1	3	11.31	7	0	3	11.04	6	0	3	11.45	9	1	3	9.93

Suppose that a quality medical practitioner would like to use the proposed repetitive sampling plan for Weibull distribution under indeterminacy to ensure the mean ratio of daily new deaths at least 60 days using the truncated life test for 60 days. Let the producer’s risk be 10% at *μ*_*N*_/*μ*_0*N*_ =1.1 and the consumer’s risk is 10%. From Table 11, with a = 1.0, $\tilde{\beta }=0.10$ and $\tilde{\alpha }=0.10$ for the repetitive sampling plan, it could be found that the plan parameters are c_1_ = 58 c_2_ = 66 and ASN = 191.67. Therefore, the plan could be implemented as follows: selecting a random sample of 114 patients from the arrived lot of patients, and doing the truncated life test for 60 days. The proposed sampling plan will be implemented as: accept the null hypothesis *H*_0_ : *μ*_*N*_ = 0.1665 if the average ratio of daily new deaths in 60 days is less than 58, the ratio of daily deaths, but the lot should be rejected as soon as the ratio of daily new deaths exceeds 66. Otherwise, the experiment would be repeated. Table 9 shows the 56 ratios of daily new deaths before the average ratio of daily new deaths of 0.1665. Therefore, the quality medical practitioners would have accepted the arrived lot of patients.

## Conclusions

An elaborated investigation of COVID-19 data for Weibull distribution under indeterminacy using time truncated repetitive sampling plan is studied. The proposed design parameters are obtained for known values of the indeterminacy parameters. The plan parameters and corresponding tables are developed for the industrial purposes at specified indeterminacy parametric values. The proposed sampling plan is compared with the existing sampling plans. The result shows that the proposed repetitive sampling plan is more economical than the existing sampling plan. The proposed sampling plan saves time; labor and amount for experimentation, the proposed plan is recommended to apply for testing the average number of deaths due to COVID-19. Also, noticed that if the indeterminacy values increase then the average sample number is decreased. The developed repetitive sampling plan procedure is illustrated with COVID-19 data belong to Italy as an application. The proposed sampling plan can be implemented in various industries covering the packing industry, medical sciences, food industries and electronic industries. Further research can be established to extend our study to group sampling plans, multiple dependent state sampling plans, and multiple dependent state repetitive sampling plans.

## Data Availability

The data is given in the paper.

## Abbreviations

ASN: Average sample number
RASP: Repetitive acceptance sampling plan
OC: Operating characteristic
Q-Q: Quantile-quantile
